# Investigation of Carbon Fibres Reclamation by Pyrolysis Process for Their Reuse Potential

**DOI:** 10.3390/polym15030768

**Published:** 2023-02-02

**Authors:** Stefania Termine, Valentina Naxaki, Dionisis Semitekolos, Aikaterini-Flora Trompeta, Massimo Rovere, Alberto Tagliaferro, Costas Charitidis

**Affiliations:** 1 Research Lab of Advanced, Composite, Nano Materials & Nanotechnology (R-NanoLab), School of Chemical Engineering, National Technical University of Athens, 9 Heroon, Polytechniou St., Zografos, 15780 Athens, Greece; 2 Department of Applied Science and Technology, Politecnico di Torino, Corso Duca degli Abruzzi 24, 10129 Turin, Italy

**Keywords:** carbon fibres, CFRPs, thermosets, pyrolysis, post-pyrolysis, recycling, reclamation

## Abstract

During Carbon Fibre Reinforced Polymers (CFRPs) manufacturing, large quantities of scrap are being produced and usually disposed to landfill or incinerated, resulting in a high environmental impact. Furthermore, CFRP parts that have been damaged or reached their end-of-life, follow the same disposal route and because of this, not only the environment is affected, but also high added-value materials, such as carbon fibres (CFs) are lost without further valorisation. Several recycling technologies have been suggested, such as pyrolysis, to retrieve the CF reinforcement from the CFRPs. However, pyrolysis produces CFs that have residual resin and pyrolytic carbon at their surface. In order to retrieve clean long fibres, oxidation treatment in high temperatures is required. The oxidation treatment, however, has a high impact on the mechanical properties of the reclaimed CFs; therefore, an optimised pyrolysis procedure of CFRPs and post-pyrolysis treatment of reclaimed fibres (rCFs) is required. In this study, CFRPs have been subjected to pyrolysis to investigate the reclamation of CF fabrics in their primal form. The temperature of 550 °C was selected as the optimum processing temperature for the investigated composites. A parametric study on the post-pyrolysis treatment was performed in order to remove the residues from the fabrics and at the same time to investigate the CFs reusability, in terms of their mechanical and surface properties.

## 1. Introduction

The demand on manufacturing and application of carbon fibre reinforced polymers (CFRPs) is rapidly growing and anticipated to increase the Compound Annual Growth Rate (CAGR) by 6% until 2030 [1]. The CFRPs are widely used in the automotive, marine, renewable energy, and construction sectors [2]. Composites are so widely used due to their advantageous properties such as high strength-to-weight ratio, fatigue resistance, and suitability for usage in harsh environmental conditions, due to their very good corrosion resistance. However, CFRP structures present some drawbacks; their properties and performance are highly dependent on the number of CF fabric layers (Vf) used, their orientation in ply level, and the polymer’s matrix characteristics [3]. When external loads are applied, such as compression forces, phenomena like fibre and matrix breakage, delamination between plies, and/or fibre buckling can occur, leading to reduction of performance or even breakage of the composite structure [4,5]. Production of composite structures constantly increases; however, at the same time, large quantities of scrap are being produced during manufacturing and, in most cases, disposed to landfill or incinerated, leading to severe environmental impacts [6].

The main recycling methods of thermoset CFRPs for the reclamation of the reinforcement are based on thermal [7] and/or chemical treatments [8]. Mechanical recycling does not separate the fibres from the matrix, and mostly crushes the composites into smaller fractions, such as granules and fine powder, that can be exploited mostly as fillers in other applications [9]. The chemicals or supercritical conditions used sometimes during chemical recycling can decompose the polymeric matrix into smaller low-molecular weight polymers or molecules. Despite the fact that the mechanical properties can be maintained up to 93%, harmful gases occur during this process and valorisation of liquid by-products is still a challenge [10,11]. Furthermore, the apparatus used in supercritical conditions (e.g., autoclaves) require expensive and complicated equipment, which increases the costs of recycling, as well as the post-process steps of the reclaimed fibres for their reuse [12]. The fibres are usually retrieved in wet and entangled form, therefore, they cannot be easily reused. In order to exploit the reinforcement of the CFRPs in their primal form, pyrolysis is suggested as the most promising reclamation process.

In this process, by treating the CFRPs in high temperatures under inert atmosphere, the polymeric matrix decomposes, and the fibres are left to be reclaimed. However, the decomposition of the matrix results in liquid, gaseous, and solid by-products. The produced gases and liquids will not be considered in this work, as the focus is to evaluate the fibres’ quality for their reuse in similar applications. The temperature range for such a process varies from 500 to 600 °C, where it has been proven that the mechanical integrity of the fibres is less affected [13]. However, by reducing the pyrolysis temperature, formation of solid by-products (residual char) occurs on the fibres’ surface, and, therefore, further treatment is required to receive clean fibres. The way to eliminate the char from the fibres can be achieved by surface oxidation of the reclaimed fibres (rCFs). Several studies have been performed to identify the optimum temperature for oxidation of the rCFs, and the results vary between 450 °C and 600 °C [7,14]. More specifically, it has been reported that the char can be partially removed at 500 °C and the tensile strength of the fibres was retained by 94% in this case [15].

With pyrolysis of woven CFRPs, it is possible to maintain the fabric dimensions and integrity, which can be valorised in new composites, through conventional manufacturing techniques. The challenge to be addressed focuses on the balance between the purity and integrity of the rCFs. Thus, the process parameters (temperature, time of pyrolysis) should provide clean fibres with the least mechanical and surface deterioration, to be easily reused for composites remanufacturing. In this work, CFRPs are pyrolysed and the char removal parameters are studied, while the reclaimed CF fabrics are reused in laminate composites manufacturing.

## 2. Materials and Methods

### 2.1. Pyrolysis Process for CF Fabrics Reclamation

The scrap composites material used for the woven fabric plies reclamation was provided by the automotive industry (Dallara Automobili, Parma, Italy). A composite part from an energy absorber, manufactured by compression moulding, was received with T700 CF woven fabric (Toray, Tokyo, Japan) and epoxy resin Araldite LY 556 (Huntsman, Texas, USA), V_f_ of 56%. The scrap part had a square hollow tube-like geometry. In order to be inserted in the pyrolysis furnace, as described below, its dimensions needed to be reduced; therefore, the part was mechanically cut in equal pieces, with dimensions of 6.5 cm × 10 cm.

For the pyrolysis process, a horizontal furnace (PTF 14/75/350, Protherm Laboratory Furnaces, Ankara, Turkey) was used, in which a cylindrical metal chamber was inserted (inner diameter ~7 cm), where the specimens were placed to be pyrolysed. The CFRP specimens were pre-deposited on a grade substrate, which enables the liquid by-products to be removed during the process. Inert atmosphere was achieved with the supply of N_2_ gas (Evrippos Gases, Chalkída, Greece) with 300 mL/min flow. Initial pyrolysis trials were performed at 500 °C for 3 h; however, the resulting product was difficult to handle and the layers could not be separated. Therefore, the pyrolysis temperature was increased to 550 °C, with 25 °C/min temperature increase step, and the isothermal residence time was doubled to 6 h. Afterwards, the furnace was left to cool down to room temperature and the reclaimed product was received. The pyrolysis specimens will be referred to as Pyr-550.

### 2.2. Post-Pyrolysis Treatments and Characterisation

For the post-pyrolysis trials, the same apparatus was used; the reclaimed material was oxidized in atmospheric conditions, leaving unsealed the inlet and outlet of the chamber. To study the char removal from the fibres, a parametric study was followed: two treatment temperatures were selected, at 400 °C and 500 °C and the oxidation time was set to 10, 20, 30, 40, and 50 min at each temperature. Consequently, the reclaimed layers from the pyrolysis process underwent oxidation treatment. The post-pyrolysis specimens will be referred to with the Temperature-Time naming; i.e., 4-10 will be the name for fabrics treated at 400 °C for 10 min. The same naming applies for specimens treated at 500 °C.

The reclaimed fabrics from pyrolysis and the fibres after post-pyrolysis were weighted to evaluate the mass removal of the formed char. Scanning Electron Microscopy (TM3030 Plus, Hitachi, Tokyo, Japan) was used to assess the fibre’s morphology and detect any residues.

Due to the possible thermal oxidation of the fibres during the post-pyrolysis, wettability assessment was performed for the evaluation of the affinity with the epoxy resin. The dynamic wettability testing that was performed, simulates the impregnation procedure during composites re-manufacturing through vacuum infusion [16]. A prototype contact angle apparatus was used, and CF bundles retrieved by the post-pyrolysed fabrics were stabilized on its stage. An epoxy resin droplet was deposited on the mounted CF bundles from an orifice of defined diameter (d = 3 ± 1 mm). High resolution cameras were used to record the spreading of epoxy droplets on the surface of the fibres. Subsequently, frames were selected to measure the droplets length to quantify the spreading time. The spreading length was measured every 20, 40, 80, 100, and 110 sec.

Post-treated fibres were evaluated by Raman Spectroscopy (inVia Reflex, Renishaw, Hong Kong, China), using a laser of 532 nm and ×20 magnification, to evaluate the oxidation process in a structural level and identify any sp^2^ distortions on the carbon fibre lattice.

### 2.3. Composites Manufacturing and Testing

Two composite laminated panels were manufactured via vacuum infusion process, by combining layers of 380 g/m^2^ Carbon Fabric (Toray T700 8000d 12, Tokyo, Japan) with epoxy resin (Araldite LY 556, Huntsman, Texas, USA). The panels were cured at 80 °C for 4 h and post cured at 120 °C for 4 h. The composite panel was pyrolysed (specimens of 23 cm × 9.3 cm) and post-pyrolysed, following the optimum conditions, as described in Section 3.1. The reclaimed fabric patches were used to re-manufacture the r-CFRP plate.

**Figure 1:**
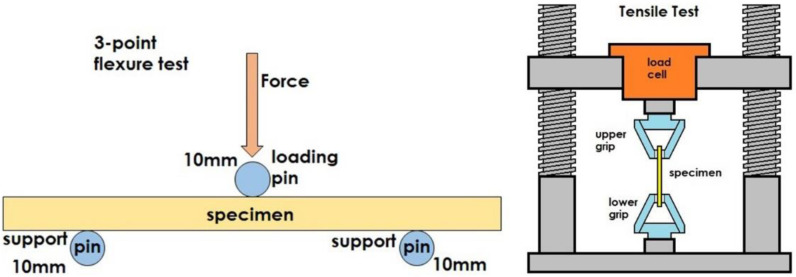
Mechanical testing schematic. (**Left**) 3-point bending, (**Right**) tensile.

Both the reference panel and the rCFRP were cut to specimens through water jetting, for mechanical testing (tensile and flexural). Tensile and flexural tests were performed to 5 specimens in an electromechanical Universal Testing Machine (WDW-50, TE Jihan testing Equipment, Jinan, China) with a maximum load of 100 kN, according to standards ASTM D638-02 [17] and ASTM D790 [18], respectively. The tensile strength is calculated according to:

(1) $$F^{\mathrm{tu}}=\frac{P_{max}}{A}$$

where, F^tu^ =ultimate tensile strength, (Mpa), *P*_max_ = maximum load prior to failure, (N), A = average cross-sectional area, (mm^2^), while the flexural strength is calculated by:

(2) $$\sigma_{f}=\frac{3\;P\;L}{2\;b\;d^{2}}$$

where, *σ_f_* = flexural stress, (Mpa), *P* = load, (N), L = support span, (mm), *b* = width of beam tested, (mm), *d* = depth of beam tested, (mm). In Figure 1, the 3-point testing and tensile schematic of the testing methodology is shown.

The reference specimens are named as Pr_sample (Pristine), i.e., Pr_a. Pr_b, etc., and the specimens with post pyrolysis treated fabrics are named Re_sample (Recycled), i.e., Re_a, Re_b, etc.

**Figure 2:**
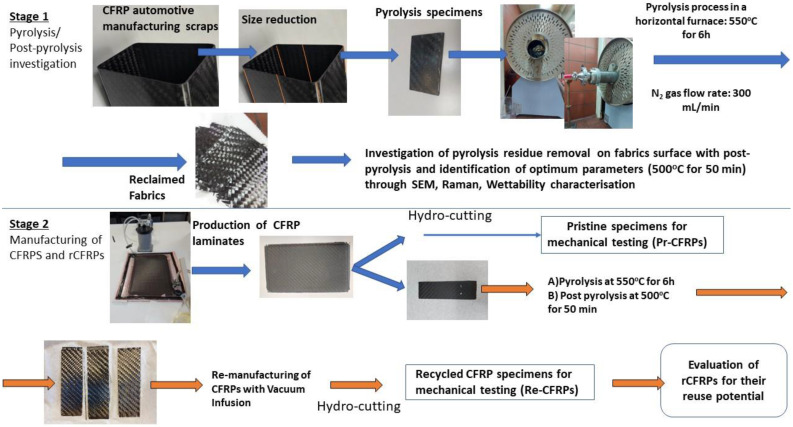
Graphical representation of the methodology followed.

In Figure 2, a graphical representation of the methodology used in this study is shown. In Stage 1, the material preparation, pyrolysis, and post-pyrolysis investigation can be observed, while in Stage 2, the CFRP manufacturing (both reference and recycled) and mechanical assessment methodology is presented.

## 3. Results

### 3.1. Pyrolysis Process

**Figure 3:**
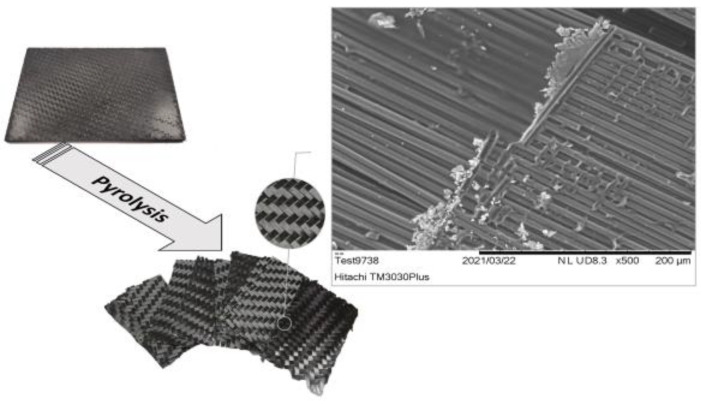
Images of composite specimen used for pyrolysis trials and reclaimed CF fabrics. (Up right: SEM image of CFs after pyrolysis at 550 °C for 6 h.)

After 6 h of pyrolysis, the fabric plies were successfully separated (Figure 3). The char formed by the resin decomposition on the surface of the fabrics was evident. To evaluate the residues on the fibres, SEM analysis was performed. A fibre bundle from the fabric was separated and placed on SEM holders. Residues creating patterns from the above fibre layers, transversal on the fibres, were observed. In other areas, where the fibres were exposed, the residue amount was significantly less.

### 3.2. Post-Pyrolysis Process

According to the literature [14,19], formation of pyrolytic carbon occurs during pyrolysis of epoxy resin. It has been proved that the removal of the char can be achieved with treatment of the fibres in thermal oxidative environment. However, oxidation can lead to reduction of properties on the fibres; therefore, a parametric study was performed to define the optimum temperature and time for this process [19].

**Figure 4:**
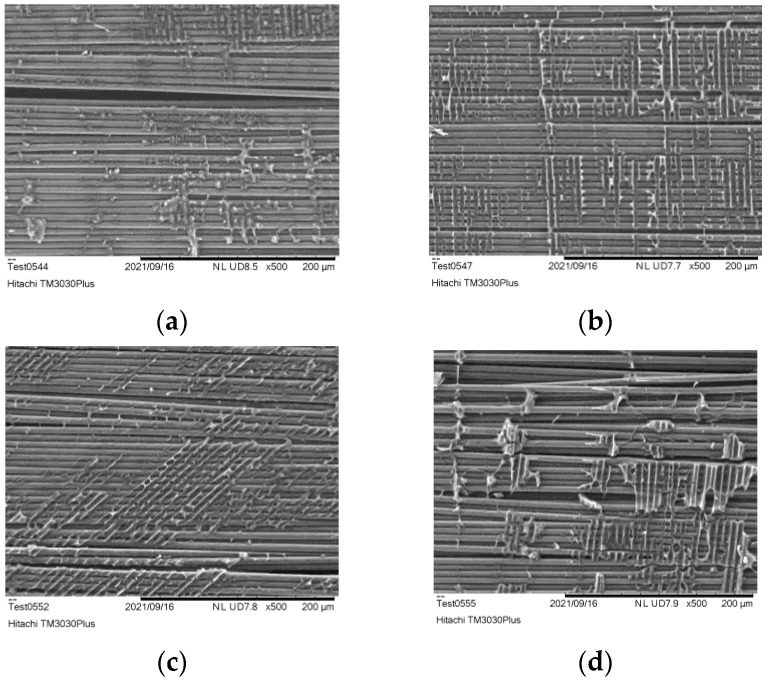

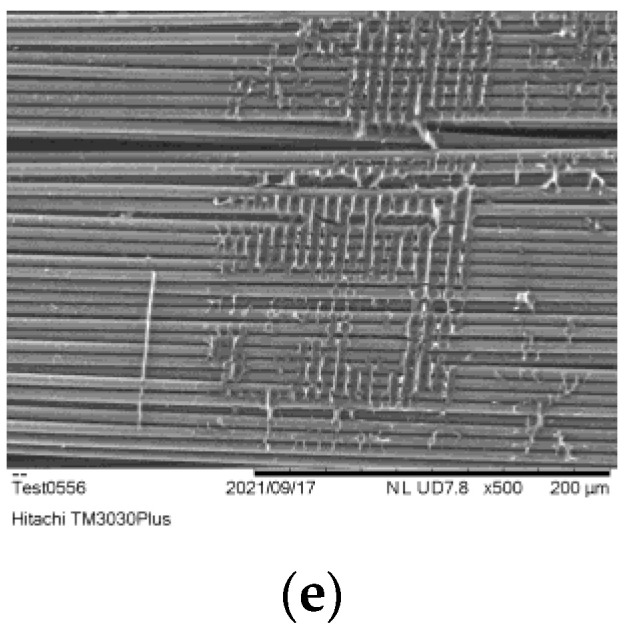
Surface analysis of CFs after post pyrolysis at 400 °C for (**a**) 10 min, (**b**) 20 min, (**c**) 30 min, (**d**) 40 min, (**e**) 50 min.

In Figure 4, SEM analysis of the post pyrolysis at 400 °C exhibited several areas where residues were still present. The less residues are observed after 40 min of treatment, without any significant change by increasing further the post-pyrolysis time.

**Figure 5:**
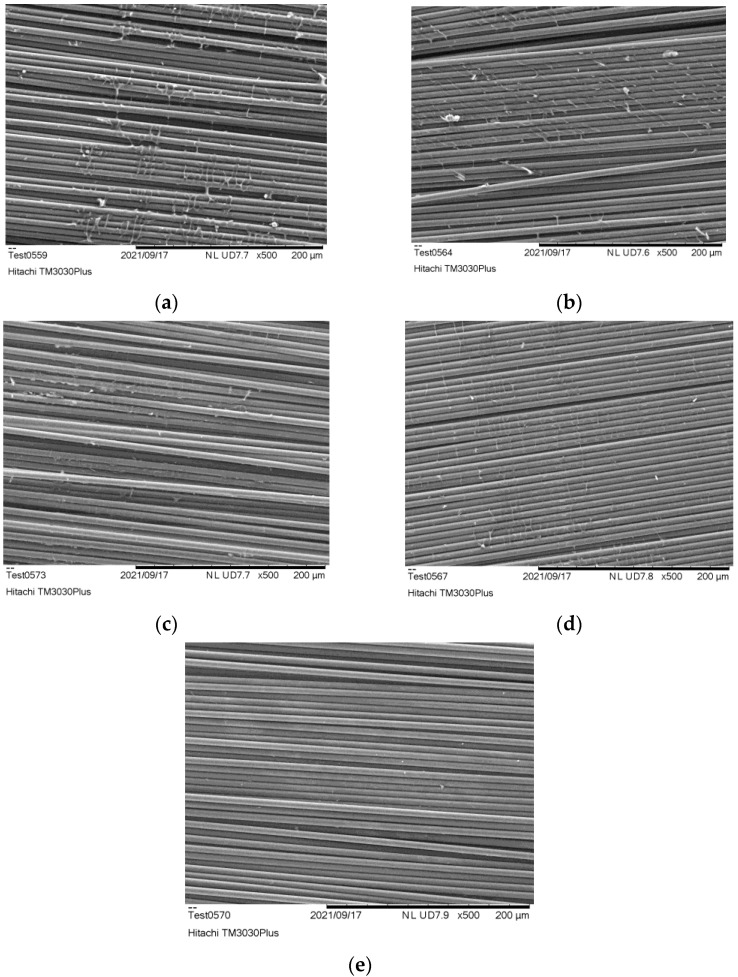
Surface analysis of CFs after post pyrolysis 500 °C for (**a**) 10 min, (**b**) 20 min, (**c**) 30 min, (**d**) 40 min, (**e**) 50 min.

When treating the fabrics at 500 °C, the amount of residue significantly decreases (Figure 5). There are still areas where residue exists, even though the treatment lasted 30 min. The optimum results are observed for a treatment lasting 50 min, where the fibres appear residue free.

To calculate the residue loss percentage from the oxidation applied, the fabrics were weighted prior and after each post-pyrolysis trials. The material loss corresponds to the mass that is being removed by the oxidation process (second process step). The weight baseline corresponds to the weight of the fabric as received from pyrolysis (mass of fibres and mass of char), which is Pyr_550. After post-pyrolysis process (for each condition), the specimen is weighed again. The difference in weight corresponds to the mass loss due to post-pyrolysis, which reflects the removal of the formed char. By calculating the weight loss percentages, the effectiveness of the post-pyrolysis conditions can be evaluated. The results are shown in Table 1.

As can be seen from Table 1, the treatment at 400 °C does not effectively remove the formed residue from the fibres, since the weight loss is negligible. On the other hand, there is a constant increase in the residue weight loss when treating the specimens at 500 °C, with the maximum loss observed after 50 min, indicating an efficient removal of the residue mass.

### 3.3. Affinity Evaluation of Post Pyrolysed Fibres with Matrix

**Figure 6:**
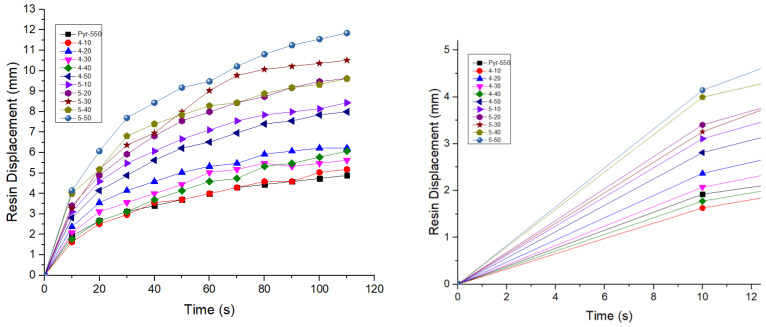
Resin spreadability evaluation on post-pyrolysis treated fibres until 120 s (**Left**), linear part until 10 s (**Right**).

The spreading behaviour of the resin on the fibre’s surface can be observed in Figure 6. In Table 2, the spreading rate from all specimens was calculated for the first 10 s of the measurement, which corresponds to the slope of the linear part of the graph. It can be observed that the rate increases with the oxidation treatment. For the 400 °C treatment, an increase of 47% is reached at 50 min, while for the 500 °C, the spreading rate has been doubled, compared to the Pyr_550 specimen.

Evidently, the fibres treated at 500 °C, exhibit not only higher spreading rates, but also the highest total resin displacement during all time intervals. This could be attributed to the effective removal of the char on the most surface area of the fibres.’

Therefore, from the above surface characterisations, it can be concluded that the optimum conditions to remove the pyrolytic residue from the surface of the fabrics is an oxidative treatment at 500 °C, with a 50 min process duration.

### 3.4. Raman Spectroscopy Analysis of Post-Pyrolysis Fibres

**Figure 7:**
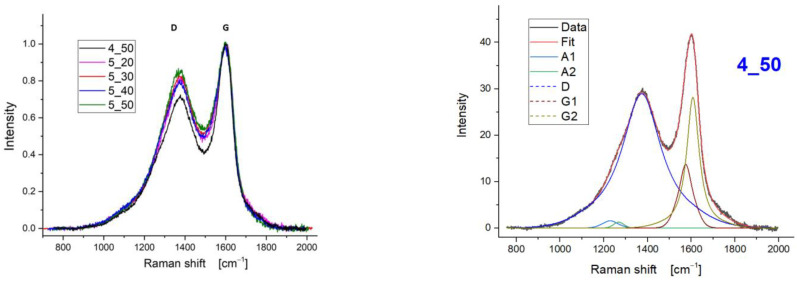
Raman spectra of treated CFs (**left**) and fitting approach on 4_50 specimen (**right**).

A specific methodology for the interpretation of the Raman results was followed. Initially, a screening of the Raman spectra in the region 900–1800 cm^−1^ was carried out. The received spectra were normalized to the G peak height to better evidence the differences in shape and they are presented in Figure 7 (left). The spectral shapes are typical for partially disordered carbons [20] with the D and G regions clearly detectable but not fully separated. All spectra were fitted with the same set of peaks (Figure 7, right), namely: A1, A2, D, G1, G2. The two peaks labelled A1 and A2 can be attributed to topological effects due to the non-uniform growth from the various C sp^2^ sites [21]. The D peak is related to the presence of sp^2^ region boundaries [20,21,22] and as such is loosely related with the size of sp^2^ coordinated regions. As clearly evidenced by Figure 7 (right), a single D component having the peak position in the range 1372–1376 cm^−1^ is sufficient to properly fit the spectra (blue line). This result is achieved by using the Gaussian–Lorentzian mixed line shape described in Tagliaferro et al. [20]. On the other end, the G peak, related to the stretching vibration of sp^2^ coordinated carbon–carbon bonds, has two components (that we will label G1 (dark red line) and G2 (green line) for the sake of simplicity, again modelled with Gaussian–Lorentzian mixed line shapes [20]). G1 (at lower wavenumbers–peaking in the range 1572–1575 cm^−1^) is attributed to the more ordered regions [21] while G2 (at higher wavelengths–peaking in the range 1608–1613 cm^−1^) arises in the presence of distortions like bond angle ones [20].

The I_D_/I_G_ ratio is also calculated to provide insights on the disorder of the sp^2^ coordinated carbon bonds [23]. In Table 3, the I_D_/I_G_ ratios are extracted from the normalised analysis of the received spectra, as mentioned in the methodology section.

**Figure 8:**
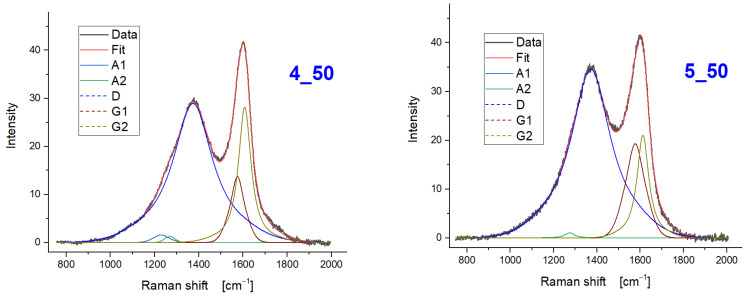
Raman spectra of post-pyrolysed carbon fibres: (**left**) 400 °C for 50 min, (**right**) 500 °C for 50 min.

From the investigation of the impact of the two treatment temperatures by comparing the Raman spectra of the fibres with the same treatment time (i.e., 4_50 and 5_50) (Figure 8), it can be summarized that:

1. The I_D_/I_G_ ratio is higher for the samples treated at higher temperature;
2. The contribution of the A1 and A2 peaks decreases at higher temperature;
3. G2 compared to G1 is more relevant at the lower temperature.

These results point towards an increase of the local order (smaller A peaks and higher G1/G2 intensity ratio) but at the same time at a slightly lower average size of the ordered regions. It can be hypothesized that single bonds receive enough thermal energy to reach a more ordered configuration; however, the energy is not large enough to bring about even a short-range reorganization. This might lead to the creation of additional boundaries as the rigidity of the network does not allow to accommodate the new situation.

**Figure 9:**
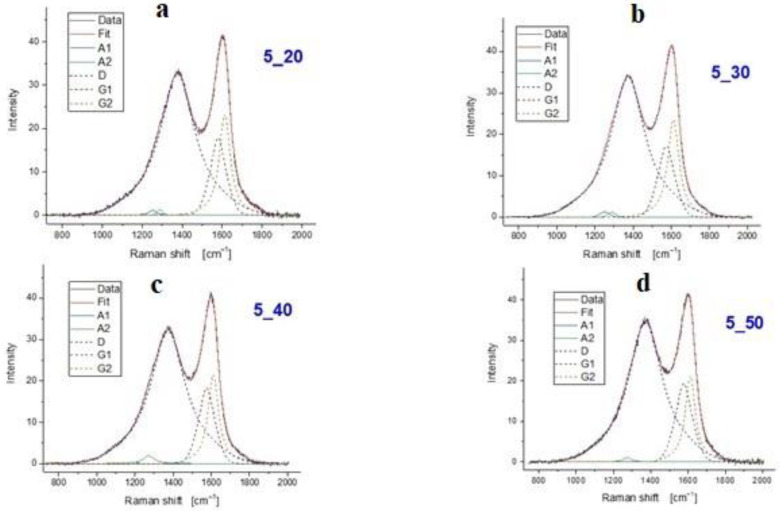
Raman spectra of post-pyrolysed carbon fibres in isothermal conditions (500 °C): (**a**) for 20 min, (**b**) for 30 min, (**c**) for 40 min, and (**d**) for 50 min.

Regarding the effect of time treatment in isothermal conditions, by comparing the spectra of the 550 °C set (Figure 9), it can be observed that:

1. The relevance of A peaks fades when the treatment time is increased;
2. The G1/G2 intensity ratio increases with treatment time;
3. I_D_/I_G_ is slightly increasing with treatment time.

### 3.5. Mechanical Evaluation of r-CFRPs

**Figure 10:**
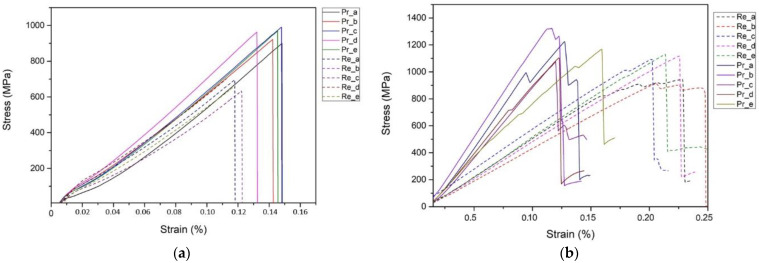
Mechanical evaluation of r-CFRPs; (**a**) Tensile Stress–Strain comparative graph; (**b**) Flexural Stress–Strain comparative graph.

The mechanical testing results are presented in Figure 10. The results of composites made with reference fibres are depicted in Figure 10a as continuous lines, while the treated fibres with dashed lines. Evidently, due to the thermal treatment of fibres, their tensile strength dwindled by 29.8% compared to the reference. The same pattern is followed for flexural strength. Recycled carbon fibre composites’ flexural strength decreased by 11.7%. In Table 4, the average values of the maximum strength are presented.

Tensile strength in CFRPs is a fibre dominant property that is related to a) the Young modulus of the fibre and b) the ability to transfer stresses from matrix to fibres. In the case of the reclaimed fibres, these conditions are reduced due to the thermal treatment of fibres. Especially for the adhesion between fibre and matrix, the interface is much weaker than on the reference samples, due to the loss of sizing during thermal treatment and its arbitrary oxidation; oxidation is also affecting the Young modulus.

Flexural strength is not a fundamental material property; it is the result of a material’s basic tensile, compressive, and shear properties working together. When flexural loading is applied to a specimen, all three basic stress states of the material are induced. The order in which the three basic stresses reach their limiting values determines material failure. It is common practice to reduce the shear stress component in the specimen to simplify the stress state by having the specimen support span (l) long enough, relative to the specimen thickness (t) (shear stress is independent of specimen length but bending, and, hence, the tensile and compressive; stress is not). The maximum tensile and compressive stresses will be equivalent if the specimen is symmetrical about the midplane of its cross section (e.g., rectangular, as in our example). Thus, which strength value is lower determines whether the specimen fails in tension or compression. The compressive strength of most composites is lower, and the specimen will fail at the compression surface. Under compressive loading, the most common failure modes are fibre shear buckling and brooming, which are caused by fibre misalignment and is highly dependent on matrix non-linearity [24,25].

Those findings can support the decrease in tensile strength of recycled CFRPs’ and the greater decrease of tensile strength compared to flexural strength.

## 4. Conclusions

In this work, pyrolysis process was carried out to study the reclamation of reinforcing fabrics from CFRPs and a parametric study was applied to identify the optimum temperature and time needed to obtain free-of-residue product in oxidative conditions. The obtained fibres were characterized in terms of their surface morphology, structural integrity, and affinity with epoxy matrix, for possible reuse as r-CFRP. The morphological analysis proved that an oxidative treatment at 400 °C is not sufficient to provide clean fibres, even after 50 min. Therefore, the temperature was increased to 500 °C, maintaining same process duration, while the results showed an increase in residue removal yield. Despite the increased temperature, the Raman analysis confirmed that the increase of the I_D_/I_G_ ratio was negligible, revealing that the CF structure remained intact. Wettability assessment showed better affinity with the epoxy matrix when the fibres were post-treated at 500 °C for 50 min. Therefore, for manufacturing the recycled CFRP, composite specimens were pyrolyzed at 550 °C for 6 h and post-pyrolysed at 500 °C for 50 min and then used for re-manufacturing. The mechanical assessment of r-CFRPs revealed a decrease in the properties. Tensile strength was reduced by 30%, while flexural strength by 12%. As a conclusion, this pyrolysis method can be applied for the reclamation of CF fabrics, rendering them viable for reuse, since they maintain their original form and geometry. Applications where flexural load is mainly applied can be considered for the r-CFRPs. Considering the current strict regulations in the automotive and construction sector due to safety reasons, recycled raw materials can be used for non-structural, aesthetical, or architectural applications. The use of recycled materials promotes a cost-efficient and environmentally friendly method to increase the presence of these advanced materials in every-day applications (e.g., home and personal care, packaging).

## Figures and Tables

**Table 1:** Weight loss percentage on fibres after post-pyrolysis treatment.

Sample (Temperature- Exposure Time)	Weight Loss (%)	Sample (Temperature- Exposure Time)	Weight Loss (%)
4-10 400 °C–10 min	0.00	5-10 500 °C–10 min	2.70
4-20 400 °C–20 min	0.22	5-20 500 °C–20 min	2.85
4-30 400 °C–30 min	0.50	5-30 500 °C–30 min	5.41
4-40 400 °C–40 min	0.54	5-40 500 °C–40 min	5.64
4-50 400 °C–50 min	1.26	5-50 500 °C–50 min	6.42

**Table 2:** Spreading rate of resin on the specimen’s surface.

Specimen	Spreading Rate (mm/s)	Specimen	Spreading Rate (mm/s)
Pyr_550	0.19	5_10	0.31
4_10	0.16	5_20	0.34
4_20	0.23	5_30	0.32
4_30	0.20	5_40	0.39
4_40	0.17	5_50	0.41
4_50	0.28		

**Table 3:** Raman I_D_/I_G_ ratio from the post-pyrolysed fibres studied.

Specimen	I_D_/I_G_
4_50	2.07
5_20	2.23
5_30	2.20
5_40	2.29
5_50	2.31

**Table 4:** Measured tensile and flexural strength of reference and r-CFRPs.

Specimen	Average Tensile Strength (ΜPa)	Average Flexural Strength (ΜPa)
Pristine CFRPs	948 ± 32	1179 ± 88
Recycled CFRPs	665 ± 28	1041 ± 93

## Data Availability

The data presented in this study are available on request from the corresponding author. The data are not publicly available according to the SMARTFAN IPRs.
